# Clinical Predictors of Dysphagia Recovery After Stroke: A Systematic Review

**DOI:** 10.1007/s00455-022-10443-3

**Published:** 2022-04-20

**Authors:** Pamela D’Netto, Anna Rumbach, Katrina Dunn, Emma Finch

**Affiliations:** 1 School of Health and Rehabilitation Sciences, The University of Queensland, St Lucia, Brisbane, QLD 4072 Australia; 2 Centre for Functioning and Health Research, Metro South Health, Brisbane, Australia; 3 Speech Pathology Department, Princess Alexandra Hospital, Metro South Health, Brisbane, Australia; 4 Speech Pathology Department, Ipswich Hospital, West Moreton Health, Ipswich, Australia

**Keywords:** Dysphagia, Stroke, Recovery, Prediction, Systematic review

## Abstract

Oropharyngeal dysphagia is common post-stroke and can have serious consequences for patients. Understanding dysphagia recovery is critically important to inform prognostication and support patients and professionals with care planning. This systematic review was undertaken to identify clinical predictors of dysphagia recovery post-stroke. Online databases (EMBASE, Scopus, Web of Science, PubMed, CINAHL, and Cochrane) were searched for studies reporting longitudinal swallowing recovery in adults post-stroke. Dysphagia recovery was defined as improvement measured on a clinical swallowing scale or upgrade in oral and/or enteral feeding status by the end of the follow-up period. The search strategy returned 6598 studies from which 87 studies went through full-text screening, and 19 studies were included that met the eligibility criteria. Age, airway compromise identified on instrumental assessment, dysphagia severity, bilateral lesions, and stroke severity were identified as predictors of persistent dysphagia and negative recovery in multiple logistic regression analysis. The available literature was predominated by retrospective data, and comparison of outcomes was limited by methodological differences across the studies in terms of the choice of assessment, measure of recovery, and period of follow-up. Future prospective research is warranted with increased representation of haemorrhagic strokes and uniform use of standardized scales of swallowing function.

## Introduction

Stroke is a common cause of major disability, with a global incidence of over 13 million people annually [1]. Oropharyngeal dysphagia is prominent across the continuum of stroke recovery [2]. Estimates of dysphagia prevalence vary according to the sensitivity of assessment measures employed and range from 8.1–45% of patients following stroke [3]. A recent study of dysphagia following ischaemic stroke suggests incidence may be declining due to improved stroke prevention, acute reperfusion therapies, and standardized care for patients admitted to stroke units [4]. Irrespective of this, more than 1 in 5 patients experience dysphagia on admission, with approximately 50% of these cases continuing to experience dysphagia at hospital discharge [4]. Post-stroke dysphagia can cause dehydration, malnutrition, and increased risk of pulmonary compromise [5, 6]. Furthermore, a dysphagia diagnosis is correlated with increased hospital costs and higher rates of institutionalization and morality [4, 6].

Due to the multiple and serious sequelae of post-stroke dysphagia, understanding the clinical course and pattern of recovery in this population is critical. Studies investigating predictors of swallowing recovery provide important information to assist clinicians with prognostication, care planning, and supportive counselling for patients and families [7]. The early identification of patients with good potential for swallowing recovery may influence decisions around the need for alternative feeding methods, such as nasogastric (NG) and percutaneous endoscopic gastrostomy (PEG) tubes [8]. Guidelines for commencing enteral nutrition acutely post-stroke include measures such as duration and severity of dysphagia [9, 10], therefore the ability to predict the probability of dysphagia recovery will guide clinician’s therapeutic decisions [11]. Additionally, accurate prediction of dysphagia recovery can support decisions regarding timing and destination of discharge [8].

Analysis of the clinical predictors of dysphagia recovery is underrepresented in the literature. There has been one previous systematic review conducted in this area by Wilmskoetter, Herbert, and Bonilha [12], which examined predictors associated with gastrostomy tube removal in patients with dysphagia after stroke. They focused on swallow recovery as the underlying cause for tube removal. Low-level evidence from retrospective studies indicated the absence of aspiration on instrumental assessment appeared to be a strong predictor of tube removal. However, a critical limitation of the 2017 review was the exclusion of patients post-stroke requiring NG tubes or other compensation such as texture-modified diets. Approximately 5% of patients admitted post-stroke require long-term gastrostomy tubes [13]; however, up to 45% of stroke patients may have some level of dysphagia [3]. Consequently, there is a need to review the current state of evidence for dysphagia recovery in all patients with stroke beyond only patients with gastrostomies.

Therefore, we sought to systematically review and evaluate the evidence for clinical predictors of dysphagia recovery post-stroke. Our objective was to identify and analyse published studies reporting longitudinal swallowing recovery following ischaemic or haemorrhagic stroke. Dysphagia recovery was denoted by reduced severity of dysphagia or change in feeding status, including commencing oral intake after NG or PEG feeding and upgrade of oral diet.

## Methods

This review was registered on the PROSPERO International Registry of Systematic Reviews (registration number: CRD42020173166). The review was conducted using the PRISMA guidelines [14].

### Information Sources

Electronic databases including (1) EMBASE; (2) Scopus; (3) Web of Science; (4) PubMed; (5) CINAHL; and (6) Cochrane, were systematically searched in March 2020 with the assistance of a university librarian. The same search was repeated in July 2021 to include new references. Additional manual searching of references lists from full-text studies was also completed.

### Search Strategy

The following terms were included in the search strategy across all databases: “swallow” (swallow* OR dysphagia OR "deglutition disorders” [All Fields] OR "deglutition disorders"[MeSH]) AND "cerebrovascular accident" (cerebrovascular accident OR stroke) AND “predict” (predict* OR prognosis OR recovery OR outcome OR convalescence OR acute). No additional filters were used during database searches.

### Study Selection

Studies were eligible if they were published in English and included patients ≥ 18 years old; with isachemic or haemorrhagic infarct; and oropharyngeal dysphagia confirmed via clinical or instrumental swallow assessment. Instrumental assessment was completed within the acute period or early subacute period post-stroke (median ≤ 30 days post-onset) to analyse severity of dysphagia. The outcome of interest was clinical factors associated with dysphagia recovery including independent predictors of recovery on multivariate regression. Dysphagia recovery was defined as reduced severity measured on a clinical scale (e.g. Functional Oral Intake Scale (FOIS)) [15], or change in feeding status (e.g. commencing oral intake after enteral feeding and/ or upgrade of oral diet) by the end of the follow-up period. This review did not investigate the influence of swallowing therapy, including acupuncture, drug therapy, behavioural interventions, electrical stimulation, physical simulation, and transcranial stimulation on dysphagia recovery post-stroke, which has been reviewed elsewhere [16]. Articles that focused on the outcome of swallowing therapy post-stroke were excluded. Studies that included a paediatric population, dysphagia of mixed aetiology, and mixed neurological cohorts were excluded. Articles that focused on assessment for risk of aspiration only (i.e. dysphagia screening), predictors of dysphagia incidence, and predictors of incidence or recovery of post-stroke pneumonia were excluded. Literature, scoping and systematic reviews, editorials, conference abstracts, research posters, and opinion papers were also excluded. The final selection of included articles was based on consensus of all authors.

### Data Analysis

Two authors (PD and KD or AR) independently reviewed each article during title and abstract screening, full-text review, data extraction, and quality assessment using the Covidence^©^ platform (https://www.covidence.org). Covidence^©^ is a free, web-based tool used to manage references and data from systematic reviews and allows reviewer’s decision to be blinded until consensus is required. Extracted data included study aims, design, patient age and gender, stroke type, measures of dysphagia, predictor variables considered, and key findings. Data were exported to Microsoft Excel where final quality analysis was completed. Meta-analysis was deemed not appropriate because of the heterogeneity of methodology and outcomes measures across the included studies.

### Quality Assessment

Methodological quality was assessed using a modified version of the McMaster Critical Review Form [17]. Questions were categorically scored as “yes” or “no,” and following consensus review categorical ratings were converted to numerical scores (1 = yes, 0 = no). Scores were assigned to questions grouped into the following domains: (1) Study purpose and literature review; (2) Sample characteristics (selection bias) including participant details, referral source, ethics procedures, and size justification; (3) Reporting of drop-outs (attrition bias); (4) Outcome measures including validity and reliability; (5) Reporting of results; and (6) Reporting of conclusions. As the studies included did not focus on swallowing therapy, quality ratings for description of intervention, contamination, and co-intervention (performance bias) were excluded from the original McMaster tool. All retrospective study designs were scored 1 for reporting of drop-outs. To score 1 for validity and reliability of outcome measures, studies needed to use measures with published psychometric properties. The maximum available score was 11.

## Results

**Fig. 1:**
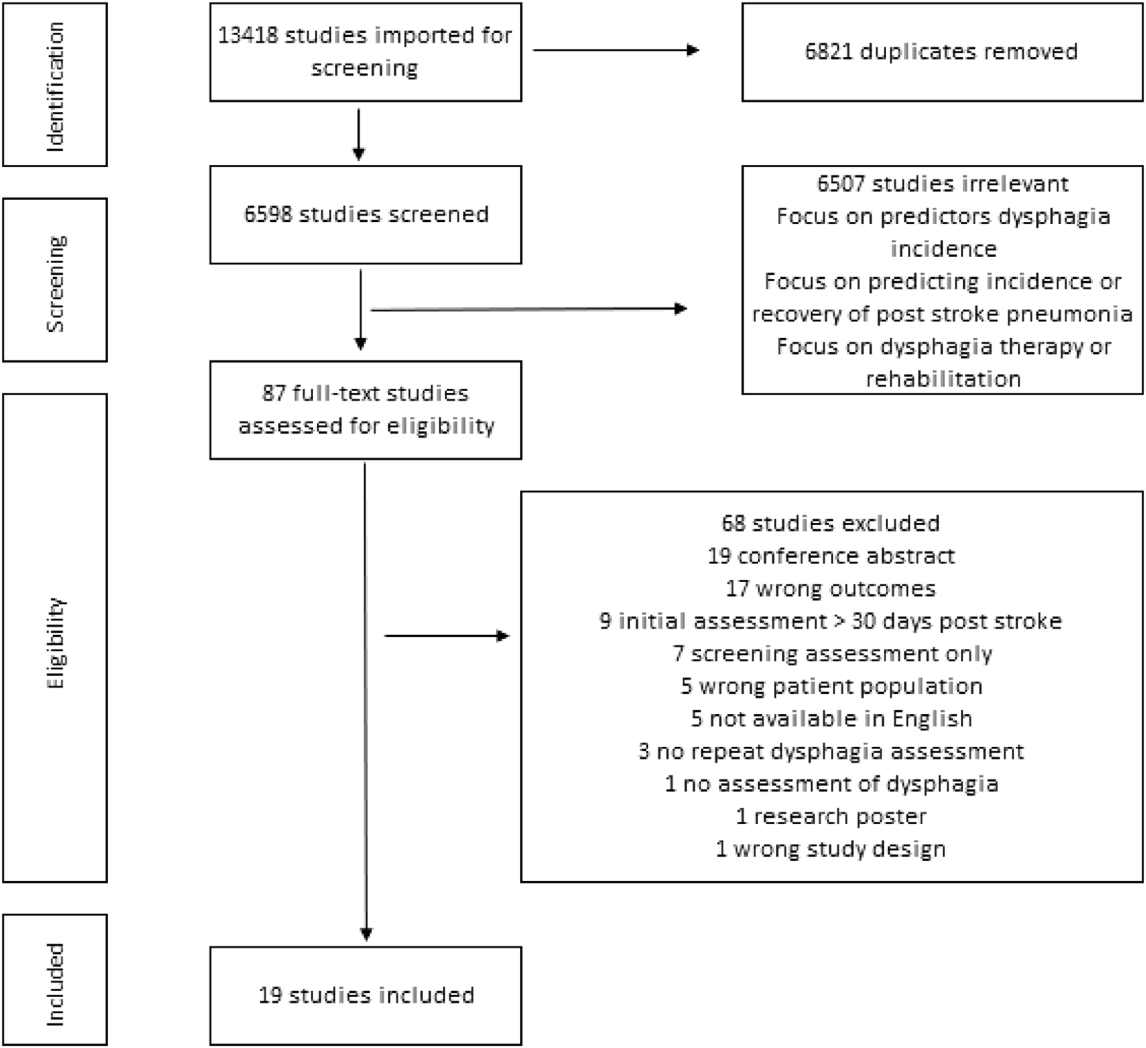
PRISMA flowchart for included studies

The study selection process is shown in Fig. 1. Electronic searches identified 11,670 studies in March 2020 and a further 1748 studies were added in July 2021 for a total of 13,418 studies (EMBASE 4430, Scopus 3041, Web of Science 2554, PubMed 2401, CINAHL 984, and Cochrane 8) matching the search criteria. After removal of duplicates, title and abstract screening were completed and 6507 studies were excluded. Full-text review was conducted on 87 studies. No additional studies were added from manual screening of the reference lists. The final analysis was completed on 19 studies matching the aim of this review. Inter-rater reliability following full-text screening was calculated on Covidence^©^ (κ = 0.51). Discrepancies were resolved through consensus from another author in the team (AR and/or EF), at each step of the screening process so as to not exclude studies prematurely, leading to potentially higher levels of conflicts as ‘maybe’ was an option in the screening process. If there was any doubt regarding inclusion, the study was put through to the next phase of screening. This may have accounted for the lower agreement.

Table 1 summarises the study aims, design, participant characteristics, outcome measures, and findings of the included studies. A variety of study designs matched our criteria including twelve retrospective cohort studies [8, 18–28], four prospective cohort studies [5, 29–31], and three studies describing the development of a prognostic model [32–34].

**Table 1:** Summary of aims and key findings

Study	Aim	Design	Sample size	Age (mean)^a^	Gender (% male)^a^	Stroke type (*n* =)	Measure of recovery	Rate of recovery^a^	Predictors/factors considered	Key findings
Calvo et al. [26]	Explore factors and predictors associated with the complete recovery of oral feeding in post-stroke patients with enteral nutrition feeding	Retrospective cohort study	163	75.8	49.1	107 IS 53 ICH 3 both	Removal of enteral feeding tube	61.9% resumed oral intake	Gender Age FIM cognitive scores FIM motor scores Time from stroke to admission Stroke location BLB swallow trigger BLB signs of aspiration	• Patients without any signs of aspiration during the water swallow test had a higher probability of resuming complete oral feeding • Patients between 73 and 79 years of age, evidence of aspiration and/or penetration, and presence of pharyngeal residue during FEES had a lower probability of returning to complete oral feeding
Choi et al. [25]	To evaluate the longitudinal changes with a kinematic analysis of VFSS in subacute stroke	Retrospective cohort study	69	67.0	62	51 IS 18 ICH	Diet at follow-up VFSS	30% change from tube feeding to oral diet at follow-up VFSS	Change in epiglottic folding angle; Hyoid displacement; and Vocal cord displacement -measured on VFSS	•Higher epiglottic folding angles may be associated with swallowing function recovery and suitability for oral feeding in subacute stroke patients
Crisan et al. [18]	Determine predictors of early recovery of functional swallow in patients who had PEG placement	Retrospective cohort study	32	–	44	17 IS 15 ICH	Removal of PEG	62.5% did not remain PEG dependent	Age Side of stroke	• Patient age as a continuous variable is a significant predictor of recovering swallowing ability
De Stefano et al. [27]	To understand the factors contributing to the severity of oropharyngeal dysphagia and its persistence in the subacute phase of stroke	Retrospective cohort study	54	75.1	55	–	Change in ASHA- NOMS and PAS	–	Age Stroke location Stroke type NIHSS Tube feeding on admission DRS PAS ASHA-NOMS	•NIHSS > 12, PAS 6–8, tube feeding on admission and haemorrhagic stroke were associated with persistent dysphagia (ASHA-NOMS 1–4) in the subacute phase •Stroke location and patient’s age were not associated with prognosis of dysphagia
Galovic et al. [32]	Describe the Predictive Swallowing Score (PRESS), for predicting the recovery of functional swallowing after ischaemic stroke	Development/ validation of a prognostic model	279	–	–	IS only	Change in FOIS score	70% FOIS > 5	Initial FOIS Frontal operculum lesion Risk of aspiration on initial CSE (measured with the any 2 test) Age Admission NIHSS	• Five predictors remained in the final simplified model: initial impairment of oral intake lesion of the frontal operculum, initial risk of aspiration, age, and NIHSS score at admission
Ickenstein et al. [29]	Null hypothesis of this study was an early prediction of severe/chronic dysphagia within 72 h of acute stroke isn't sufficiently possible	Prospective cohort study	114	–	–	103 IS 11 ICH	Change in ASHA- NOMS at day 90	–	Age Gender Stroke category Stroke location ASHA NOMS level 1–3 within 72 h PAS 5–8 within 72 h	• Clinical swallowing examination combined with a swallowing endoscopy can reasonably predict continued supplemental tube feeding
Kim et al. [19]	Investigate the association between infract location and the duration of dysphagia recovery	Retrospective cohort study	59	65.8	50.8	IS only	Recovery to stage 3 diet	–	Stroke location	• Patients with lesions in the posterior limb of the internal capsule and/ or in the caudate nucleus had longer recovery times
Kumar et al. [8]	Analyse clinical, radiologic, and treatment-related factors that are independently associated with persistent dysphagia at hospital discharge	Retrospective cohort study	323	75.9	41	IS only	Change in oral diet	30% resumption of full or mildly modified diet	Gender Intubation Bilateral infarcts Acute stroke therapy NIHSS ≥ 12 Dysarthria Nil aspiration on VFSS Positive aspiration on VFSS	• Presence of aspiration, severe dysarthria, baseline NIHSS score ≥ 12, bilateral infarcts, and intubation during hospitalisation are independently associated with persistent dysphagia at hospital discharge in acute ischaemic stroke
Lee et al. [20]	Demonstrate associating factors regarding NG removal in patients with dysphagia after stroke	Retrospective cohort study	138	–	–	IS only	Removal of NG	–	Stroke location	• Non-brainstem group: NIHSS and mRS showed a positive correlation with NG removal time; MMSE and MBI showed a negative correlation with NG removal time • Brainstem group: none of the factors studied showed correlation • For both groups: change in mean PAS was significantly different according to NG removal status
Lee et al. [33]	Develop a machine learning based prognostic model for long-term swallowing outcomes	Development/ validation of a prognostic model	137	68.7	50.4	IS only	Nil feeding tube or diet modification at 6 months	–	Tube feeding recommended at initial VFSS Male sex Clinical dysphagia scale score ≥ 20 Bilateral lesions at the corona radiata, basal ganglia or internal capsule Sever white matter hyperintensities	• The duration of post-stroke dysphagia significantly differed by tube feeding status; clinical dysphagia scale; sex; severe white matter hyperintensities and bilateral lesions at the corona radiata; basal ganglia or internal capsule
Lee et al. [28]	To investigate hyoid kinematic features for poor swallowing prognosis	Retrospective cohort study	36	–	66.7	IS only	Nil feeding tube or diet modification at 6 months	–	Hyoid displacement and velocity during VFSS	• Reduced horizontal hyoid displacement and velocity, and decreased angle of hyoid movement for the early phase of swallowing is observed in patients with poor swallowing prognosis
Lin et al. [21]	To study the applicability of NIHSS in early prediction of prognosis of post-stroke dysphagia	Retrospective cohort study	165	71 (median)	66.7	IS only	Change in FOIS score	56.4% FOIS at discharge higher than FOIS at admission	Sex Age Old lesions on MRI Comorbidities In-hospital pneumonia Admission FOIS Speech therapy interval Days of hospital stay Each item of the NIHSS	• High NIHSS scores of facial palsy and language/aphasia showed significantly negative effects on the early improvement in dysphagia
Mann et al. [5]	To study the prognosis of swallowing function over the first 6 months after acute stroke	Prospective cohort study	128	–	64	117 IS 10 ICH 1 both	Return to pre-stroke diet	87% return to pre-stroke diet	Age Sex Stroke category Stroke syndrome Barthel Index Abnormality on CSE Abnormality on VFSS Communication abnormality	• Delayed oral transit on VFSS was an independent predictor of failure to achieve the patient’s pre-stroke diet at 6 months
Oh et al. [31]	To explore if outcome of post-stroke swallowing disturbance could be affected by single-nucleotide polymorphisms	Prospective cohort study	206	63.8	66	119 IS 81 ICH	Return to oral intake within 3 months	64% returned to oral intake at 3 months	Age Stroke location NIHSS score Tracheostomy insertion Single-nucleotide polymorphisms related to an increasing risk of dysphagia in the elderly or related to affect post-stroke motor recovery	• Age, stroke location (multiple), NIHSS score, and tracheostomy insertion was associated with increased risk of poor swallowing outcome • No single gene was associated with increased risk of poor swallowing at 3 months
Schroeder et al. [22]	Determine specific neurological predictors associated with dysphagia and recovery in acute stroke patients	Retrospective cohort study	65	67	–	IS only	Diet at discharge and change in PAS on repeat VFSS	75% oral diet at discharge	Hemispatial neglect;aphasia	• Hemispatial neglect was associated with non-oral intake on initial swallowing evaluation only • Aphasia was not associated with swallowing outcomes
Seo et al. [23]	Evaluate longitudinal changes of swallowing in stroke patients with aspiration	Retrospective cohort study	28	66.9	67	23 IS 5 ICH	Resolved aspiration on VFSS	50% resolved aspiration	Temporal and spatial elements related to hyoid and epiglottic movement	• Delayed swallowing triggering at initial VFSS is associated with poor recovery from aspiration in subacute stroke patients
Smithard et al. [30]	Assess the frequency and natural history of swallowing problems following an acute stroke	Prospective cohort study	121	79 (median)	44	59 IS 22 ICH 40 not classified	Change in risk of aspiration on CSE or VFSS	89% no identified risk of aspiration at 6 months post-stroke	Gender Side of weakness Presence of neglect Incontinence Abbreviated mental test score Barthel score Side of stroke	• No significant difference in clinical features were identified between those with new and persistent dysphagia to predict unsafe swallow at day 28
Wang et al. [34]	To identify the factors for dysphagia recovery and develop a model that predicts dysphagia trajectory	Development/ validation of a prognostic model	485	62.1	70.3	IS only	Change in FOIS score	71.2% FOIS = 7 at 30 days	Age Stroke type Stroke location Stroke treatment NIHSS Glasgow Coma Scale Barthel Index Body Mass Index Dysarthria FOIS	•Predictors at day 7 included age, FOIS, NIHSS, hemispheric stroke, and brainstem stroke on admission •Day 14 predictors included age, FOIS, and NIHSS on admission •Day 30 predictors included age, FOIS, NIHSS, bilateral stroke, and body mass index on admission
Wilmskoetter et al. [24]	Assess clinical factors and their relationship with the acute recovery of post-stroke dysphagia	Retrospective cohort study	44	67.5	48	IS only	Change in FOIS score	70% FOIS 4–7 at discharge	Age Comorbidity score Length of stay MBSImp components Total lesion volume	• Damage to the left hemisphere in the 1) superior frontal gyrus, 2) dorsal anterior cingulate gyrus, 3) hypothalamus, and 4) nucleus accumbens, were significantly associated with less improved oral intake • Age independently predicted change in FOIS

*ASHA-NOMS* American Speech-Language-Hearing Association National Outcome Measurement System, *BLB* Bilancio Logopedico Berve, *CSE* Clinical Swallowing Examination, *FIM* Functional Impairment Measure, *FOIS* Functional Oral Intake Scale, *ICH* intercranial Haemorrhage, *IS* Ischaemic Stroke, *MBI* modified Barthel Index, *MMSE* Mini Mental State, *mRS* modified Rankin Scale, *NIHSS* National Institute of Health Stroke Scale, *NG* Nasogastric Tube, *PEG* Percutaneous Endoscopic Gastrostomy, *PAS* Penetration-Aspiration Scale, *VFSS* Videofluoroscopic Swallow Study

^a^Missing data or reported as reported within group mean or percentage

### Participant Characteristics

The total number of included patients across all studies was 2618 (Table 1). Participants from Lee et al. [28] were not counted in total participants as this study reported a sub-analysis of data from another included study, Lee et al. [33]. More than half of the included studies recruited patients with ischaemic stroke only [8, 20–22, 24, 32–34]; therefore, 86% of patients from all included studies had ischaemic stroke. An overwhelming majority of patients were first ever stroke, but two studies [21, 22] included patients with history of previous stroke (*n* = 92; 3.5%). Different classification systems for stroke location were used across the included studies (e.g. grouped by vascular territory, supratentorial or infratentorial, clinical syndrome; exact lesion location and laterality) making it difficult to analyse this variable. In particular, numbers of patients with brainstem lesions (*n* = 220; 8%), an important location for swallowing pathophysiology due to the direct impact on bulbar cranial nerves, was only clearly reported in 7 studies [8, 20, 30–32, 34, 35].

Mean age of patients across the included studies was 62.9 years; however, five studies [5, 20, 28, 29, 32] reported age as a within group mean or percentage and could not be included in this calculation. Gender ranged from 44 to 71% males in the sample. The age and gender distribution of included patients are consistent with global incidence [1].

### Measures of Dysphagia and Recovery

Table 2 outlines the variety of clinical and instrumental assessments used across the included studies. All studies utilized instrumental assessment with eligible patients. Videofluoroscopic Swallow Study (VFSS) was favoured by most authors, while four studies used Fibreoptic Endoscopic Evaluation of Swallow (FEES) [26, 27, 29, 32]. Eight studies repeated instrumental assessment with all participants [19, 20, 23, 25, 27, 28, 31, 33]. The interval between repeat instrumental assessments ranged from 1 week to 3 months, while the total follow-up period ranged from time of discharge from acute hospital to six months post-stroke. The majority of studies used clinician reported measures at initial and follow-up assessment, but one study [29] used patient-reported outcome at 90-day follow-up.

**Table 2:** Measures of dysphagia

Study	Measure of dysphagia severity	Instrumental assessment (%)	Period of follow-up	Follow up provided
Calvo et al. [26]	BLB FEES	85	Until discharge from inpatient rehabilitation	BLB performed at discharge
Choi et al. [25]	VDS score ASHA NOMS VFSS	100	Not clearly stated	100% completed repeat VFSS at least 1 week after initial VFSS
Crisan et al. [18]	CSE VFSS	34	Until discharge from inpatient rehabilitation	Not clearly stated
De Stefano et al. [27]	DRS FEES ASHA NOMS PAS	100	Until discharge from subacute rehabilitation	100% completed repeat FEES after 15–20 days and 60 days from initial assessment
Galovic et al. [32]	50 ml water swallow test Any 2 scale Parramatta Hospitals Assessment of Dysphagia FOIS FEES	As deemed clinically necessary	≥ 4 weeks	Clinical evaluation at baseline and 7 days Phone interview > 4 weeks post-onset
Ickenstein et al. [29]	ASHA NOMS FEES	100	3 months	Phone interview at 90 days
Kim et al. [19]	VFSS	100	Time to prescription of dysphagia diet stage 3	100% completed VFSS performed at intervals of one week
Kumar et al. [8]	CSE VFSS	36	Until discharge from acute hospital	CSE at discharge
Lee et al. [20]	VFSS PAS	100	Until recovery or discharge from inpatient rehabilitation	100% completed VFSS at follow- up every 2 weeks
Lee et al. [33]	CDS VFSS	100	6 months	Interval of VFSS was < 4 weeks during the initial phase and gradually prolonged to 1–3 months
Lee et al. [28]	CDS VFSS	100	6 months	100% completed VFSS at outpatient follow- up review up to 6 months post-stroke
Lin et al. [21]	FOIS VFSS	As deemed clinically necessary	Until discharge from inpatient rehabilitation	FOIS at discharge
Mann et al. [5]	CSE VFSS	100	6 months	60% completed repeat VFSS
Oh et al. [31]	MASA FOIS VFSS PAS EAT-10	100	3 months	100% repeated the battery of assessments at 3 months
Schroeder et al. [22]	CSE VFSS PAS	55	Until discharge from inpatient rehabilitation	28% completed repeat VFSS
Seo et al. [23]	VDS score ASHA NOMS VFSS	100	Not clearly stated	100% completed repeat VFSS 2–4 weeks after initial
Smithard et al. [30]	CSE VFSS	79	6 months	85% completed repeat VFSS at 28 days
Wang et al. [34]	Water swallow test CSE VFSS or FEES FOIS	As deemed clinically necessary	30 days	Repeat assessment to score FOIS at day 7, 14 and 30
Wilmskoetter et al. [24]	FOIS VFSS	100	Until discharge from acute hospital	SLP assessment before discharge

*ASHA-NOMS* American Speech-Language-Hearing Association National Outcome Measurement System, *BLB* Bilancio Logopedico Berve, *CDS* Clinical Dysphagia Scale, *CSE* Clinical Swallowing Examination, *DRS* Dysphagia Risk Scores, *EAT-10* Eating Assessment Tool, *FEES* Fibreoptic Endoscopic Evaluation of Swallowing, *FOIS *Functional Oral Intake Scale, *MASA* Mann Assessment of Swallowing Ability, *PAS* Penetration-Aspiration Scale, *VDS* Videofluoroscopic Dysphagia Scale, *VFSS* Videofluoroscopic Swallow Study

Dysphagia recovery was broadly defined as change in feeding status or change in severity on a validated dysphagia scale across all studies. There was heterogeneity in the scales used to measure dysphagia severity. The Functional Oral Intake Scale (FOIS) was the most common, employed in five studies [21, 24, 31, 32, 34]. Rate of recovery measured by change in FOIS ranged from 56 to 71% (see Table 1). Other dysphagia scales used to measure swallowing recovery included the American Speech-Language-Hearing Association National Outcome Measure System (ASHA-NOMS) [23, 25, 27, 29]; the Penetration-Aspiration Scale (PAS) [22, 27]; and the Videofluoroscopic Dysphagia Scale (VDS) [23, 25]. Change in aspiration status on VFSS reported without reference to a validated scale was used as a measure of recovery by Smithard et al. [30].

Swallowing recovery was also described as change in feeding status; however, heterogeneity was present in use of this outcome measure across the studies. Return to oral intake following removal of an enteral feeding tube was defined as significant recovery in four studies [18, 20, 26, 31]; however, the authors did not specify the level of textured modified diet prescribed when enteral feeding was ceased. A further study defined recovery as time to first prescription of rice porridge following tube feeding only [19]; however, it was unclear whether patients were still supplemented with top-up enteral feeds. Three studies reported dysphagia recovery as return to premorbid diet without modification [5, 28, 33]. Finally, Kumar et al. [8] accepted resumption of a full diet or oral intake with one restricted consistency (e.g. soft diet) as recovered swallowing function. Due to the heterogeneity of outcome for feeding status across the studies rate of recovery was not uniformly reported and ranged from 30 to 87% (Table 1).

### Clinical Predictors of Dysphagia Recovery

A variety of clinical predictor variables for dysphagia recovery were investigated across the included studies (see Table 1). Logistic regression was used to identify independent predictors of dysphagia recovery in eight of the included studies (Table 3) [5, 8, 18, 21, 24, 26, 29, 31]. In these studies, predictors were categorized as positive for favorable recovery or negative if predicating persistent dysphagia. Additionally, three studies [32–34] used regression in the development of multivariate prognostic models of dysphagia recovery (Table 4). The remaining eight studies [19, 20, 22, 23, 25, 27, 28, 30] investigated clinical factors associated with dysphagia recovery without regression analysis.

**Table 3:** Clinical predictors of dysphagia recovery post-stroke from multiple logistic regression

Study	Sample	Outcome examined	Predictor variables considered	Positive predictors of recovery	Negative predictors of recovery	AUC	OR	95% CI	*p* =
Calvo et al. [26]	139	Complete recovery of oral feeding at discharge defined as removal of enteral feeding support	Gender Age FIM cognitive scores FIM motor scores Time from stroke to admission Stroke location BLB swallow trigger BLB signs of aspiration FEES aspiration/ penetration FEES residue	No signs of aspiration on BLB			3.57	1.07–11.89	0.03
					Age 73–79 years		0.096	0.01–0.58	0.01
					Aspiration/ or penetration on FEES		0.22	0.07–0.72	0.01
					Residue on FEES		0.14	0.04–0.43	< 0.01
Crisan et al. [18]	34	Recovery of swallow function following PEG placement defined as discontinuation of PEG dependence	Age Side of stroke	Younger Age			0.89	0.82–0.98	0.016
				Left sided stroke			15.15	1.32–173.34	0.016
Ickenstein et al. [29]	114	Prediction of 90-day outcome	Age Gender Stroke category Stroke location ASHA NOMS level 1–3 within 72 h PAS 5–8 within 72 h		Combined tube feeding dependency (ASHA NOMS 1–3) and aspiration on FEES (PAS 5–8) within 72 h of admission	0.782	11.8	0.036–0.096	< 0.001
Kumar et al. [8]	323	Presence of dysphagia at discharge from acute hospital, defined as any swallowing impairment leading to ≥ 2 dietary modification	Gender Intubation Bilateral infarcts Stroke Treatment NIHSS ≥ 12 Dysarthria Nil aspiration on VFSS Positive aspiration on VFSS		Intubation	0.892	2.857	1.106–7.38	0.0301
					Bilateral infract		3.725	1.33–10.43	0.0123
					NIHSS ≥ 12		2.510	1.189–5.296	0.0157
					Dysarthria		3.4	1.572–7.355	0.0019
					Positive aspiration on VFSS		10.50	3.351–32.955	< 0.001
Lin et al. [21]	165	Early improvement of dysphagia defined as positive value of: discharge FOIS—admission FOIS	Sex Age Old lesions on MRI Comorbidities In-hospital pneumonia Admission FOIS Speech therapy interval Days of hospital stay Each item of the NIHSS		NIHSS item 4—facial palsy	0.731	0.484	0.279–0.838	0.0096
					NIHSS item 9—language/aphasia	0.714	0.562	0.321–0.982	0.043
Mann et al. [5]	15	Different diet at 6 months after stroke	Age Sex Stroke category Stroke syndrome Barthel index Abnormality on CSE Abnormality on VFSS Communication abnormality		Delayed oral transit on VFSS		32	4.1–26.1	
Oh et al. [31]	206	Increased risk of nil per oral status at 3 months	Age Stroke location NIHSS score Tracheostomy insertion Single-nucleotide polymorphisms related to an increasing risk of dysphagia in the elderly or related to affect post-stroke motor recovery		Age	0.774	1.05	1.02–1.09	0.0027
					Stroke location		3.65	1.15–11.57	0.00276
					NIHSS score		1.07	1.01–1.13	0.0149
					Tracheostomy insertion		26.1	6.5–104.13	< 0.001
Wilmskoetter et al. [24]	44	FOIS change from first to last speech pathology encounter	Age Comorbidity score Length of stay MBSImp components Total lesion volume		Age		β = 0.03		0.04

*ASHA-NOMS* American Speech-Language-Hearing Association National Outcome Measurement System, *BLB* Bilancio Logopedico Berve, *CSE* clinical swallowing examination, *FIM* Functional Impairment Measure, *FEES* Fibreoptic Endoscopic Evaluation of Swallowing, *FOIS* Functional Oral Intake Scale, *NIHSS* National Institute of Health Stroke Scale, *PEG* percutaneous endoscopic gastrostomy, *PAS* Penetration-Aspiration Scale, *VFSS* Videofluoroscopic Swallow Study

**Table 4:** Prognostic models of dysphagia recovery post-stroke

Study	*N* =	Outcome	Predictor variables included	Model type	Validation	Model discrimination	Model calibration
Galovic et al. [32]	153 derivation cohort 126 validation cohort	Primary: persistence of severely impaired oral intake (FOIS score > 5) at follow-up on day 7 and day 30 Secondary: return to pre-stroke diet	Initial FOIS Frontal operculum lesion Risk of aspiration on initial CSE (measured with the any 2 test) Age Admission NIHSS	Cox proportional hazards model	Split- sample	C- statistic for predicting the recovery of oral intake on day 7 = 0.84 (95% CI 0.76–091; *p* < 0.001)	Calibration plots for day 7 and day 30 Hosmer- Lemeshow test
						C- statistic for predicting the recovery of oral intake on day 30 = 0.77 (95% CI 0.67–0.87; *p* < 0.001)	
						C- statistic for predicting return to pre-stroke diet on day 7 = 0.94 (95% CI 0.87–1.00; *p* < 0.001)	
						C- statistic for predicting return to pre-stroke diet on day 30 = 0.71 (95% CI 0.61–0.82; *p* < 0.001)	
Lee et al. [33]	137	Swallowing function at 6 months post-stroke	Tube feeding recommended at initial VFSS Male sex Clinical dysphagia scale score ≥ 20 Bilateral lesions at the corona radiata, basal ganglia or internal capsule Sever white matter hyperintensities	Machine learning—Bayesian network model	Fivefold cross validation	ROC curve = 0.802	
Wang et al. [34]	340 training set 145 validation set	To predict dysphagia recovery (FOIS = 7) at follow-up on day 7, day 14 and day 30	Age Stroke type Stroke location NIHSS GCS BI BMI FOIS	Multivariable logistic regression nomogram	Bootstrapping	C indices for prediction nomograms were: day 7: 0.847 (95% CI 0.804–0.884) day 14: 0.817 (95% CI 0.772–0.857) day 30: 0.786 (95% CI 0.739–0.829)	Calibration curve of the nomogram for the probability of day 7, day 14, and day 30 swallowing recovery

*BI* Barthel Index, *BMI* Body Mass Index, *CI* confidence interval, *CSE* clinical swallowing examination, *FOIS* Functional Oral Intake Scale, *GCS* Glasgow Coma Scale, *NIHSS* National Institute of Health Stroke Scale, *ROC* receiver operating characteristic, *VFSS* Videofluoroscopic Swallow Study

The most common finding on logistic regression was that physiological features of dysphagia identified on instrumental assessment independently predicted dysphagia recovery. Airway compromise as evidenced by penetration or aspiration on instrumental swallow exam was the only symptom identified as a negative predictor of dysphagia recovery by more than one study [8, 26, 29]. Other physiological features predictive of persistent impairment included delayed oral transit on VFSS [5] and residue post-swallow on FEES [26]. However, no instrumental features of dysphagia were carried through into prognostic models for swallowing recovery. Instead these models found significant association for functional swallowing examination scores, i.e. FOIS [32, 34] and variables from clinical swallowing examination (CSE) including risk of aspiration on the Any 2 swallow test [32] and moderate–severe dysphagia on the Clinical Dysphagia Scale (CDS ≥ 20) [33].

Stroke-related variables such as severity, location, and co-occurring impairments were also commonly identified predictors of recovery in regression analysis. The National Institute of Health Stroke Scale (NIHSS) was used to measure stroke severity in several studies, but treatment of the variable varied across studies. Early research from Kumar et al. [8] dichotomized the NIHSS score and demonstrated moderate stroke severity (NIHSS ≥ 12) was an independent predicator of poor dysphagia recovery. However, Oh et al. [31] found that total NIHSS on admission was predictive of poor recovery at 3 months post-stroke. Differences in definition of recovery, return to full or minimally modified oral diet [8] versus return to any oral diet [31], may account for this difference. Baseline NIHSS was a significant variable in two prognostic models for dysphagia recovery [32, 34] indicating stroke severity is an important determinant of swallowing prognosis within the first month post-stroke. In addition, subitems of the NIHSS measuring facial palsy and communication impairment were also found to independently predict persistent dysphagia [8, 21]. However, Kumar et al. [8] found that severe dysarthria (NIHSS item 10) was significant, while Lin et al. [21] found language/aphasia (NIHSS item 9) was predictive of negative recovery. Kumar and colleagues [8] did not include the presence of aphasia in their covariate analysis. However, Lin et al. [21] included both dysarthria and aphasia in their analysis, choosing to analyse each item of the NIHSS separately, and dysarthria did not reach significance.

No consensus was identified for stroke location as an independent predictor from multivariate logistic regression. Oh et al. [31] found lesions located at regions affecting both the supratentorial to infratentorial areas (i.e. multiple sites) was predictive of poor swallowing outcomes. Other lesion locations and stroke laterality did not reach significance in their model. This is in contrast to earlier findings from Crisan et al. [18] who found left-sided stroke to be predictive of favorable recovery; however, their results may be under powered given the small sample size (*n* = 32). Stroke location was a variable in the prognostic models of dysphagia recovery (Table 4); however, each model favored different locations including frontal operculum [32], bilateral lesions at the corona radiata; basal ganglia or internal capsule [33]; and cortical or brainstem locations [34]. Other stroke-related variables predictive of poor swallowing recovery were bilateral lesions [8, 33, 34] and severe white matter hyperintensities [33].

Age was the most investigated demographic variable. When analyzed as a continuous variable, one study reported younger age as a positive predictor of dysphagia recovery [18], while others [24, 31, 34] reported age as a negative predictor of recovery. Furthermore, age as a categorical variable was also a negative predictor of recovery [26, 32].

The final category of predictor variables identified from logistic regression in the included studies was medical interventions. Intubation and tracheostomy insertion post-stroke were negative predictors of dysphagia recovery [8, 31]. Sub-analysis of factors related to these interventions such as duration of intubation was not possible [8]. Reperfusion treatments including thrombolysis and thrombectomy were investigated by one predictive study [8] and one prognostic model [34] but did not reach significance.

### Study Quality

Study quality was assessed using the McMaster Critical Review Form for Quantitative Studies [17]. Extracted data is presented in Table 5 according to study design. Four studies [18, 21, 31, 32] achieved the maximum score of 11. All studies demonstrated strength in reporting attrition bias and most studies scored highly for purpose, literature, and results. One study [30] did not provide sufficient explanation on method for statistical analysis of results. Several studies [5, 8, 19, 20, 22, 26, 27, 30] reported outcome measures which were judged as not valid or reliable. Of these studies, four [5, 8, 19, 30] reported change in diet descriptively rather than using a standardized scale, and one study [26] reported outcomes as change in dysphagia severity on the non-validated Bilancio Logopedico Berve tool [36]. Finally, three studies [20, 22, 27] reported outcomes as change in severity based on airway compromise measured on the PAS [37]. Recent work [38, 39] has suggested that aggregating, summarizing, or simplifying PAS results has the potential to reduce the reliability or validity of the result. For the studies sighted in this review, Schroder et al. [22] reported PAS as an ordinal scale and subsequently categorized values to indicate severity. In contrast, De Stefano et al. [27] and Lee et al. [20] reported change in mean PAS for different participant groups. Given the discrepancy and the conjecture in the literature over statistical interpretation of PAS score [38], this outcome was judged as not reliable.

**Table 5:** Evaluation of study quality using the McMaster critical review form

Design/study	Purpose and Literature (-/2)	Selection Bias (-/2)	Attrition Bias (-/1)	Outcome Measures (-/2)	Results (-/2)	Conclusions (-/2)	Total (max 11)
Development/validation of a prognostic model							
Galovic et al. [32]	2	2	1	2	2	2	11
Lee et al. [33]	2	2	1	1	2	1	9
Wang et al. [34]	2	1	1	2	2	2	10
Prospective cohort studies							
Ickenstein et al. [29]	2	0	1	2	2	1	8
Oh et al. [31]	2	2	1	2	2	2	11
Mann et al. [5]	2	1	1	0	2	2	8
Smithard et al. [30]	2	1	1	0	1	0	5
Retrospective cohort studies							
Calvo et al. [26]	2	2	1	0	2	2	9
Choi et al. [25]	2	1	1	2	2	2	10
Crisan et al. [18]	2	2	1	2	2	2	11
De Stefano et al. [27]	2	1	1	0	2	2	8
Kim et al. [19]	2	2	1	0	2	1	8
Kumar et al. [8]	2	2	1	0	2	1	8
Lee et al. [20]	1	1	1	0	2	2	7
Lee et al. [28]	2	1	1	1	2	1	8
Lin et al. [21]	2	2	1	2	2	2	11
Schroeder et al. [22]	2	2	1	0	2	2	9
Seo et al. [23]	2	2	1	1	2	2	10
Wilmskoetter et al. [24]	2	1	1	2	2	2	10

## Discussion

This systematic review has examined the published evidence for clinical factors associated with and independent predictors of dysphagia recovery post-stroke. Dysphagia is a common consequence post-stroke with serious complications; therefore, accurate prediction of recovery can enable informed decision making for patients and assist clinicians to develop strategies to modify care. This review identified 19 studies that met the inclusion criteria. There was substantial heterogeneity across study design, statistical methods, and measures of dysphagia recovery. Despite this, we were able identify physiological, demographic, stroke, and treatment variables that can influence dysphagia recovery.

This work expands on the findings from Wilmskoetter, Herbert, and Bonilha [12] who systematically reviewed predictors of recovery in patients post-stroke with severe dysphagia, where recovery was defined as removal of a gastrostomy tube. The authors analyzed 6 retrospective studies and determined the absence of aspiration on VFSS was the strongest predictor for tube removal. A limitation of the previous review was that dysphagia recovery was evaluated by the single outcome of feeding tube removal. In the current review, we included studies where dysphagia recovery was denoted by change in severity on a validated dysphagia scale or change in feeding status including oral and enteral feeding. This criterion for dysphagia recovery was more sensitive to change in swallowing function as evidenced by the higher number of included papers and range of included study designs; however, the wide definition of recovery introduced considerable variability for assessment, outcome measures, and period of follow-up. We acknowledge that there is some overlap between the two criteria for recovery as many dysphagia scales include an item that reports tube or supplemental feeding. However, analysis of studies which reported recovery solely as change in feeding status revealed oral diet texture was not routinely reported following tube removal. This prohibited retrospective scoring with a dysphagia scale. Furthermore, despite all studies reporting recovery based on clinician recommendations (except [29] at follow-up only), many studies reported different end points even when using the same measure of recovery. For example, four studies used change in FOIS as an outcome measure for dysphagia recovery but each study had a different definition of successful recovery including: any positive increase in FOIS score [21]; FOIS 4–7 [24] FOIS > 5 [32]; and FOIS = 7 [34]. This type of inconsistency between the studies analyzed precluded meta-analysis.

A variety of independent predictors for dysphagia recovery were identified by the studies in the current review including demographic, stroke and treatment variables, and physiological features of dysphagia. Of these, the strongest predictor of persistent dysphagia (negative recovery) identified in logistic regression was confirmed penetration and/ or aspiration on instrumental swallowing examination [8, 26, 29]. This supports the findings of Wilmskoetter, Herbert, and Bonilha [12] who found the absence of aspiration on VFSS was a positive predictor of recovery. In contrast to the previous review [12], we were able to extend the evidence for age as a negative predictor of recovery [24, 26, 31, 32, 34]. Additionally, younger age was a positive predictor of feeding tube removal [18], supporting previous findings [40, 41], and the importance of this variable as a predictor of dysphagia recovery.

In this review, aspiration identified on FEES or VFSS was equally predictive of negative recovery. Other physiological features of dysphagia identified on instrumental assessment, including delayed oral transit on VFSS [5] and residue post-swallow on FEES [26] were also found to predict persistent dysphagia. In addition, change in temporal and spatial measurements of hyoid and epiglottis movement on VFSS were associated with dysphagia recovery [23, 25, 28]. These findings support the importance of conducting instrumental assessment in the care of patients with dysphagia post-stroke. Objective judgement of pharyngeal physiology and airway compromise is important in determining the severity of dysphagia which is associated with health outcomes and healthcare cost [40]. However, significant limitations apply to conducting instrumental assessments with all patients in the acute phase of stroke such as equipment availability, clinical stability, level of alertness, and inability to comply with instructions due to poor cognitive–communicative state [41]. Furthermore, no instrumental variables were significant in the prognostic models for swallowing recovery included in this review. We acknowledge the methodological difference between the prognostic models, but some agreement was reported for severity on initial clinical swallow exam (CSE) [32, 33] and initial FOIS score [32, 34] as significant predictors of dysphagia recovery. Therefore, clinicians should aim to utilize a standardized CSE in dysphagia management [42], such as the Mann Assessment of Swallowing Ability (MASA) [43], in combination with standardized scales of severity such as the FOIS [15] or the Dysphagia Severity-Rating Scale (DSRS) [44] when instrumental assessment is not possible. A limitation of the FOIS and the DSRS is that they do not include International Dysphagia Diet Standardisation Initiative (IDDSI) terminology [45]; however, Everton et al. [44] have recently suggested an update to the DSRS to include the IDDSI terminology which may increase the robustness of this scale for future use.

Stroke-related factors were found to predict dysphagia recovery; however, the evidence was mixed. Stroke severity as conferred by NIHSS score was the strongest stroke variable related to dysphagia recovery. Higher initial NIHSS score was associated with longer time to removal of NG in non-brainstem stroke [20]. Furthermore, NIHSS score was an independent predictor of outcome when included as a continuous variable [31, 32, 34] and in itemized analysis [8, 21] in regression analysis. NIHSS item 4 (facial palsy), 9 (language/ aphasia) [21], and 10 (dysarthria) [8] were negatively correlated with dysphagia recovery. In contrast, Schroder et al. [22] did not find an association between aphasia on admission and dysphagia recovery; however, this group did not complete regression analysis and used a different assessment of aphasia. Differences in methodology and statistical analysis between these studies [8, 21, 22] preclude a definitive finding on which type of communication disorder is more predictive of dysphagia recovery in this systematic review; however, the trend suggests communication ability post-stroke influences other aspects of function and is an important correlate for potential for recovery.

Other stroke-related factors influencing recovery identified in this review were bilateral lesions, stroke location, and severe white matter hyperintensities [33]. Evidence was available from regression analysis to suggest bilateral lesions impede swallowing recovery [8, 33, 34]. This is congruous given that swallowing musculature has been demonstrated to have bilateral cortical innervations [46]. We were unable to demonstrate consensus on lesion location as a predictor of dysphagia recovery with individual studies reporting a variety of significant variables. Stroke location was reported according to different classification systems across the included studies creating a discrepancy in analysis. In particular, brainstem lesions were individually reported in some studies but grouped with other infratentorial lesions in other studies, which may explain why this did not emerge as an independent predictor of recovery. From the available evidence, longer recovery times were reported for patients with lesions in the posterior limb of the internal capsule and the caudate nucleus [19]; however, in multivariate analysis, left-sided stroke [18], frontal operculum lesions [32] and lesions located at regions affecting both the supratentorial to infratentorial areas (i.e., multiple sites) [31] all showed significance depending on methodology and outcome investigated.

Medical treatments including intubation [8] and tracheostomy insertion [31] post-stroke were negative predictors of dysphagia recovery. This may be due to increased severity of dysphagia caused by laryngeal injuries from the tubes, mucosal damage, impaired sensation, and myopathy, in addition to neurological injuries, in stroke patients undergoing either procedure [47]. Interestingly, reperfusion treatments including thrombolysis and thrombectomy did not reach significance for the prediction of dysphagia recovery [8, 34] despite recent findings suggesting that patients undergoing thrombolysis had greater improvement of oral intake and shorter hospital stay [48]. Further research is required to fully understand the relationship between reperfusion and dysphagia outcomes [48–50] given the increasing use of these treatments [51].

Several limitations are acknowledged in the analysis provided. Firstly, 86% of patients from all included studies had ischaemic stroke, even though we did not seek to exclude patients with haemorrhagic stroke. Therefore, results of this review may not be generalizable to patients in the wider stroke population. Secondly, only a small number of prospective studies were included. The majority were retrospective and, therefore, faced inherent limitations such as accurate record keeping and convenience sampling. Third, as discussed above, methods of dysphagia assessment, measures for swallowing recovery, and period of follow-up were not uniform across the included studies. This introduced a referral bias in some studies and restricted the ability to draw conclusions and make recommendations for clinical management for this population. Future prospective studies should incorporate more patients with haemorrhagic stroke and reach agreement on a recognized, standardized measure of recovery. Furthermore, future research needs should consider multidimensional assessment that incorporates clinician recommendations from objective assessment (VFSS and FEES) using validated scales, alongside clinical assessment, and patient-reported outcome measures. The addition of patient-reported outcomes measure may reduce reporting bias as it is possible that many patients choose a diet not recommended by their clinician despite risk [52]. Finally, we acknowledge that a discussion on dysphagia recovery would ideally include analysis of predictors of recovery from swallowing therapy studies since there is an increasing number of prospective trials in this area. We have chosen to focus on clinical predictors of dysphagia recovery which we hoped would be more generalizable to the knowledge base of stroke recovery without caveats related to specificity or intensity of individual therapy programmes. Synthesis of clinical and therapy-based predictors of dysphagia recovery remains a topic to be examined in future reviews.

## Conclusion

This systematic review has identified physiological, demographic, stroke, and treatment variables that can influence dysphagia recovery after stroke. Studies reporting recovery as change in severity on a valid dysphagia scale or change in oral and/ or enteral feeding status were included. We found consensus from two or more studies for predictors of persistent dysphagia and negative recovery including penetration or aspiration identified on instrumental assessment, age, bilateral lesions, initial FOIS score, and stroke severity measured by the NIHSS. This information is vital to patients, carers, and health professionals when considering care options. Interest in this topic in the literature appears to be growing with multiple study designs identified; however, available evidence is predominated by retrospective data. Furthermore, comparison of outcomes is limited by methodological differences in the choice of assessment, measure of recovery, and period of follow-up. Future research with equal representation of stroke types and uniform use of standardized scales of swallowing function is warranted.
